# Polymorphic control in titanium dioxide particles†

**DOI:** 10.1039/d2na00390b

**Published:** 2022-11-23

**Authors:** Gabriel Quiñones Vélez, Diego Soto Nieves, Anushka Castro Vázquez, Vilmalí López-Mejías

**Affiliations:** a Department of Chemistry, University of Puerto Rico Río Piedras San Juan Puerto Rico 00931 USA vilmali.lopez@upr.edu; b Crystallization Design Institute, Molecular Sciences Research Center, University of Puerto Rico San Juan 00926 Puerto Rico USA; c Department of Accounting, University of Puerto Rico Río Piedras San Juan Puerto Rico 00931 USA

## Abstract

The hydrolysis–condensation reaction of TiO_2_ was adapted to the phase inversion temperature (PIT)-nano-emulsion method as a low energy approach to gain control over the size and phase purity of the resulting metal oxide particles. Three different PIT-nano-emulsion syntheses were designed, each one intended to isolate high purity rutile, anatase, and brookite phase particles. Three different emulsion systems were prepared, with a pH of either strongly acidic (H_2_O : HNO_3_, pH ∼0.5), moderately acidic (H_2_O : isopropanol, pH ∼4.5), or alkaline (H_2_O : NaOH, pH ∼12). PIT-nano-emulsion syntheses of the amorphous TiO_2_ particles were conducted under these conditions, resulting in average particle diameter distributions of ∼140 d nm (strongly acidic), ∼60 d nm (moderately acidic), and ∼460 d nm (alkaline). Different thermal treatments were performed on the amorphous particles obtained from the PIT-nano-emulsion syntheses. Raman spectroscopy and powder X-ray diffraction (PXRD) were employed to corroborate that the thermally treated particles under H_2_O : HNO_3_ (at 850 °C), H_2_O : NaOH (at 400 °C), and H_2_O : isopropanol (at 200 °C) yielded highly-pure rutile, anatase, and brookite phases, respectively. Herein, an experimental approach based on the PIT-nano-emulsion method is demonstrated to synthesize phase-controlled TiO_2_ particles with high purity employing fewer toxic compounds, reducing the quantity of starting materials, and with a minimum energy input, particularly for the almost elusive brookite phase.

## Abstract 2

The hydrolysis–condensation reaction of TiO_2_ was adapted to the phase inversion temperature (PIT)-nano-emulsion method, leading to the selective formation of highly-pure rutile, anatase, and brookite particles of this metal oxide.

## Graphical abstract

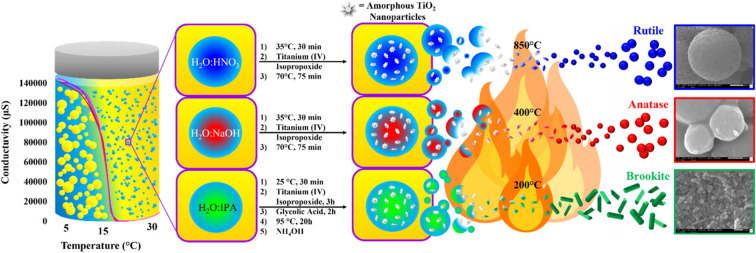

## Introduction

Nowadays, titanium dioxide (TiO_2_) is one of the most studied materials because of its promising physicochemical properties.^1–5^ Both the physical and chemical nature of this metal oxide have enabled it as a multifunctional material employed for a wide range of applications,^1–8^ such as for electrochromic devices,^9–11^ photocatalysis,^8,12,13^ photovoltaics,^14,15^ pigments,^16^ and sensing.^17,18^ The versatility of TiO_2_ is driven by its ability to crystallize as several polymorphs, such as rutile, anatase, and brookite, the three most studied phases.^2,4,5^ The three polymorphs exhibit unique properties, making one preferred over another for certain applications, such as photocatalysis, where anatase is the best candidate.^4^ When comparing these phases, rutile is the thermodynamically stable form and most abundant polymorph of the metal oxide, while anatase and brookite are metastable.^2,4–7,19–22^ Commonly, the phase transformation of anatase to rutile is irreversible at moderate to elevated temperatures.^19–22^ Although pure anatase has been isolated at relatively low calcination temperatures,^23^ using higher temperatures leads to the observation of rutile phase impurities. Moreover, brookite is the TiO_2_ polymorph that is most difficult to synthesize; thus, only scarce studies regarding synthetic routes and properties have been reported for specific applications.^4,5,24–26^ Therefore, achieving phase selectivity of the metal oxide depends on the chemical properties of each polymorph. This aspect needs to be considered when trying to isolate a specific phase of TiO_2_.

The overall performance of TiO_2_ for a given application not only depends on the crystal phase, but also on the particle size, morphology, and phase purity achieved through the synthetic method employed.^4^ Several methods for synthesizing TiO_2_ with varied size, shapes, and purity have been reported.^3,6–8,27^ Among these, sol–gel, hydrothermal, solvothermal, chemical vapor deposition, and sonochemical methods are the most utilized for this purpose.^3,27,28^ However, many of these experimental approaches require expensive precursors, large amounts of reagents, hazardous materials or expensive instrumentation, and/or produce hazardous contaminants.^3,24,27–29^ Although some of the syntheses reported have achieved highly-pure phase TiO_2_ particles at various sizes, there is still a need to improve and diversify these methods.

In previous reports, the phase inversion temperature (PIT)-nano-emulsion method has been demonstrated to successfully decrease and control the particle size of materials during their syntheses.^30,31^ The PIT method is a low-energy emulsification method that is employed for the formation of nano-emulsions without mechanical manipulation, sophisticated instrumentation, and high energy input.^32–34^ On the contrary, the PIT method uses only the chemical potential of the emulsion components (aqueous phase, oil phase, and surfactant) and high stirring under changes in temperature to achieve the resulting nano-emulsion.^34^ Here, the emulsion components are homogenized at room temperature and cooled down afterward to produce an oil-in-water (O/W) micro-emulsion. Then, the micro-emulsion is heated until reaching the PIT, in which the system changes from the O/W to an water-in-oil (W/O) nano-emulsion.^34^ Commonly, the nano-emulsions obtained through the PIT-nano-emulsion method achieve nanodroplets with average diameters in the range of 10–500 nm and relatively low polydispersity index (PDI) values.^34^ To date, the synthesis of TiO_2_ employing this method has not been reported.

In this work, the synthesis of TiO_2_ was adapted to the PIT-nano-emulsion method to confine and limit the hydrolysis–condensation reaction space into that produced by the aqueous nanodroplets after reaching the PIT. The synthesis of amorphous TiO_2_ particles was carried out inside these nanospheres to control their particle size. Three different PIT-nano-emulsion syntheses were designed, each one oriented to isolate one of the polymorphic phases of TiO_2_. The selectivity to each polymorphic phase was subject to the chemical nature of the aqueous phase from the emulsions and the thermal treatment employed. Different emulsion systems were prepared; specifically, the pH was moderately acidic [H_2_O : isopropyl alcohol (IPA)/heptane emulsion system to selectively prepare brookite], strongly acidic (H_2_O : HNO_3_/heptane emulsion system to selectively prepare rutile), or alkaline (H_2_O : NaOH/heptane emulsion system to selectively prepare anatase). Under the respective conditions and employing the minimum quantity of starting materials during syntheses, highly-pure TiO_2_ particles with well-defined morphologies were achieved and unambiguously confirmed through multiple solid-state characterization techniques. This study is intended to provide for the first time an experimental approach based on the PIT-nano-emulsion method to synthesize phase-controlled TiO_2_ particles of rutile, anatase, and brookite phases.

## Experimental section

### Materials

Nanopure water from an ARIES Filter Works Gemini High Purity water system (18.23 MΩ cm), nitric acid [HNO_3_, 70% wt] from Fisher Chemical, sodium hydroxide (NaOH, ACS Grade) from VWR® AMRESCO® (Solon, OH), isopropyl alcohol (IPA, ACS Grade) from BDH® VWR® (Radnor, PA), heptane [CH_3_(CH_2_)_5_CH_3_, anhydrous 99%] from Alfa Aesar (Ward Hill, MA), and BrijL4® [(C_20_H_42_O_5_)_*n*_, average *M*_n_ ∼362] from Sigma-Aldrich (St. Louis, MO) were employed for preparing the emulsion systems. Acetic acid glacial [C_2_H_4_O_2_, HPLC Grade] from ACS (Billerica, MA) and ethanol [C_2_H_6_O, ACS/USP Grade] from Pharmco-Aaper (Brookfield, CT) were used to prepare the titanium precursor solutions for the anatase and rutile phase syntheses. Titanium(iv) isopropoxide [C_12_H_28_O_4_Ti, 97% pure] from Sigma-Aldrich (St. Louis, MO) was employed as the titanium precursor in all the syntheses. For the isolation of the brookite phase, glycolic acid (ReagentPlus®, 99%) from Sigma-Aldrich (St. Louis, MO) and aqueous ammonia (ammonium hydroxide, NH_4_OH, 28–30%, ACS Grade) from BDH® ARISTAR® (West Chester, PA) were employed.

### TiO_2_ bulk syntheses (control)

Bulk syntheses of the rutile (H_2_O : HNO_3_), anatase (H_2_O : NaOH), and brookite (H_2_O : IPA) phases were carried out as the control groups. The experimental details can be found in the ESI.†

### H_2_O : HNO_3_/heptane emulsion system

A mixture of H_2_O : HNO_3_ was prepared separately by diluting 6 mL of nitric acid in 100 mL of nanopure water. The emulsion system was prepared by adding 11 mL of H_2_O : HNO_3_ solution, 3 mL of heptane, and 0.9 BrijL4® into a 20 mL scintillation vial.

### H_2_O : NaOH/heptane emulsion system

A mixture of H_2_O : NaOH was prepared separately by diluting 6 mL of NaOH 1 M in 100 mL of nanopure water. The emulsion system was prepared by adding 11 mL of H_2_O : NaOH solution, 3 mL of heptane, and 0.9 mL of BrijL4® into a 20 mL scintillation vial.

### H_2_O : IPA/heptane emulsion system

The emulsion system was prepared by adding 9.75 mL of nanopure water, 1.25 mL of IPA, 3 mL of heptane, and 0.9 mL of BrijL4® into a 20 mL scintillation vial.

### Phase inversion temperature (PIT) determination

For the H_2_O : HNO_3_/heptane and H_2_O : NaOH/heptane emulsion systems, the PIT was determined as follows. The respective mixture was homogenized using an IKA T10 Basic Ultra Turrax (IKA Works Inc., Wilmington, NC) for 30 s at a speed of “4” (14 450 rpm equivalent). The vial was situated in a jacketed beaker, with a 20.3 cm (8′′) stainless-steel RTD temperature probe (VWR®, VWR International). The conductivity of the emulsion was measured with a Fisherbrand Accumet BasicAB30 conductivity meter (Fisher Scientific UK, Loughborough, UK). The bath temperature was controlled with a Julabo F32-ME Refrigerated/Heating Circulator (JULABO GmbH, Seelbach, Germany). Both the vial and the bath contained magnetic stir bars stirring at 300 rpm using a VWR® Professional Hot Plate Stirrer (97042-714, VWR®, VWR International). The temperature of the emulsion was allowed to reach 2 °C in the bath before starting the measurements. The temperature profile started at 2 °C and ended at 37 °C at a heating rate of 1°C min^−1^. The conductivity of the respective mixture was recorded in 1-degree intervals.

### Titanium precursor solution

For the rutile and anatase phase particles syntheses, preparation of a titanium precursor solution was required. For this, 3 mL of ethanol, 20 drops of acetic acid glacial, and 1 mL of titanium(iv) isopropoxide were mixed inside a 20 mL beaker and sealed with parafilm.

### PIT-nano-emulsion syntheses of TiO_2_ particles

The nano-emulsion syntheses of the TiO_2_ particles were conducted in a Crystalline™ multireactor crystallization system (Technobis, Crystallization Systems, Alkmaar, Netherlands). These syntheses were tested as a proof-of-concept in 8 mL reactors inside of the Crystalline™. Each reactor can produce up to 150 mg of TiO_2_ per batch (the Crystalline™ has 8 reactors), with reproducible experimental yields ranging from 80 to 90%. Although not attempted, the reagents, pressures, and temperatures are amenable to process scale-up. The calcination of the resulting amorphous TiO_2_ from the rutile and anatase phase PIT-nano-emulsion syntheses was conducted in an Isotemp Muffler Furnace (Fisher Scientific). The experimental conditions and parameters employed to isolate the brookite phase TiO_2_ nanoparticles using the PIT-nano-emulsion synthesis described here were compared to other reported methods (ESI†) to provide evidence of the ability of the described parameters to crystallize and isolate phase-controlled TiO_2_ brookite nanoparticles with high purity employing fewer toxic compounds, reducing the quantity of starting materials, and with minimum energy input when compared to other previously employed methods.^24–26^

#### Rutile and anatase phases

The H_2_O : HNO_3_/heptane (rutile) or H_2_O : NaOH/heptane (anatase) emulsion systems were prepared and homogenized. Once the emulsion was homogenized, 2.5 mL of each were transferred to separate Crystalline™ reaction vials with a stir bar and sealed with a reflux cap. The vials were then placed in the first reactor at 5 °C (rutile) or 6 °C (anatase) for 30 min under 1250 rpm continuous stirring. Then, the vials were transferred to a second reactor at 45 °C for 30 min. Once the 30 min elapsed, 1 mL of the titanium(iv) precursor solution previously prepared was added into the reaction vials *via* a syringe and the vials were left for 75 min at 70 °C. Once this process was completed, the suspension (amorphous titania + aqueous phase) was transferred to a crucible and calcinated at 850 °C for 2 h for isolating the rutile phase. However, to isolate the anatase phase, the suspension was transferred to a crucible and calcinated at 400 °C for 24 h.

#### Brookite phase

The H_2_O : IPA/heptane emulsion system was prepared and homogenized. Once the emulsion was homogenized, 2.5 mL was transferred to a Crystalline™ reaction vial with a stir bar and sealed with a reflux cap. The vial was placed in a reactor at 5 °C for 30 min under 1250 rpm continuous stirring. Then, the temperature was raised to 25 °C and 0.5 mL of titanium(iv) isopropoxide was added *via* a syringe. The vial was left for 3 h under continuous stirring. Subsequently, 5 mL of a 1.05 M glycolic acid solution was added to the vial *via* a syringe and left for 2 h under stirring. Once the time elapsed, the temperature was raised to 95 °C and kept for 20 h without stirring. Once the process was completed, the aqueous phase with the white precipitate from the vial was transferred to a beaker and the pH was adjusted to ∼12 with NH_4_OH. The pH-adjusted suspension was transferred to a digestion bomb (243AC-T304-051716A, Parr Instrument Company, Moline, IL) and heated at 200 °C for 20 h. Finally, the product was centrifuged and washed twice with nanopure water.

### Dynamic light scattering (DLS) measurements

DLS samples were analyzed in a Malvern Panalytical Zetasizer NanoZS (Spectris PLC, Surrey, England) equipped with a He–Ne orange laser (633 nm, max 4 mW). Data was analyzed with Malvern software, version 7.12. The reaction vial from each nano-emulsion synthesis was undisturbed for 1 h prior to analysis. Aliquots of 50 μL of the supernatant from the aqueous phase were transferred and diluted in disposable polystyrol/polystyrene cuvettes (REF: 67.754, 10 × 10 × 45 mm, Sarsted, Germany) in a 1 : 40 dilution ratio with nanopure water. The refractive index used for the sample was 1.372, which corresponds to TiO_2_ in water. This value was determined by measuring an aliquot of TiO_2_ stock solution with a Mettler Toledo Refracto 30GS (Mettler Toledo, Columbus, OH).

### Raman microscopy

Raman spectra were recorded in a Thermo Scientific DXR Raman microscope, equipped with a 532 nm laser, 400 lines per nm grating, and 50 μm slit. The spectra were collected at room temperature over the range from 3400 and 100 cm^−1^ by averaging 32 scans with exposures of 3 s. The OMNIC for Dispersive Raman software version 9.2.0 was employed for data collection and analysis.

### Powder X-ray diffraction (PXRD)

The collection of powder X-ray diffractograms was performed in the transmission mode (300 K) using a Rigaku XtaLAB SuperNova X-ray diffractometer with a micro-focus Cu Kα radiation (*λ* = 1.5417 Å) source and equipped with a HyPix3000 X-ray detector (50 kV, 0.8 mA). Powder samples were mounted in MiTeGen microloops. Powder diffractograms were collected between 6 and 60° with a step of 0.01° using the Gandolfi move experiment. CrystAllis^PRO^ software v. 1.171.3920a was used to analyze the data.

### Scanning electron microscopy-energy dispersive spectroscopy (SEM-EDS)

Micrographs and X-ray microanalysis were recorded with a JEOL JSM-6480LV scanning electron microscope with an Evenhart Thomley secondary electron imagining (SEI) detector and an energy dispersive X-ray analysis (EDAX) Genesis 2000 detector. Images were taken with an acceleration voltage of 20 kV, an electron beam of 11 mm width, a spot size value of 36, SEI signal, and in the high vacuum mode.

## Results and discussion

### PIT-nano-emulsion syntheses of TiO_2_ particles

**Fig. 1:**
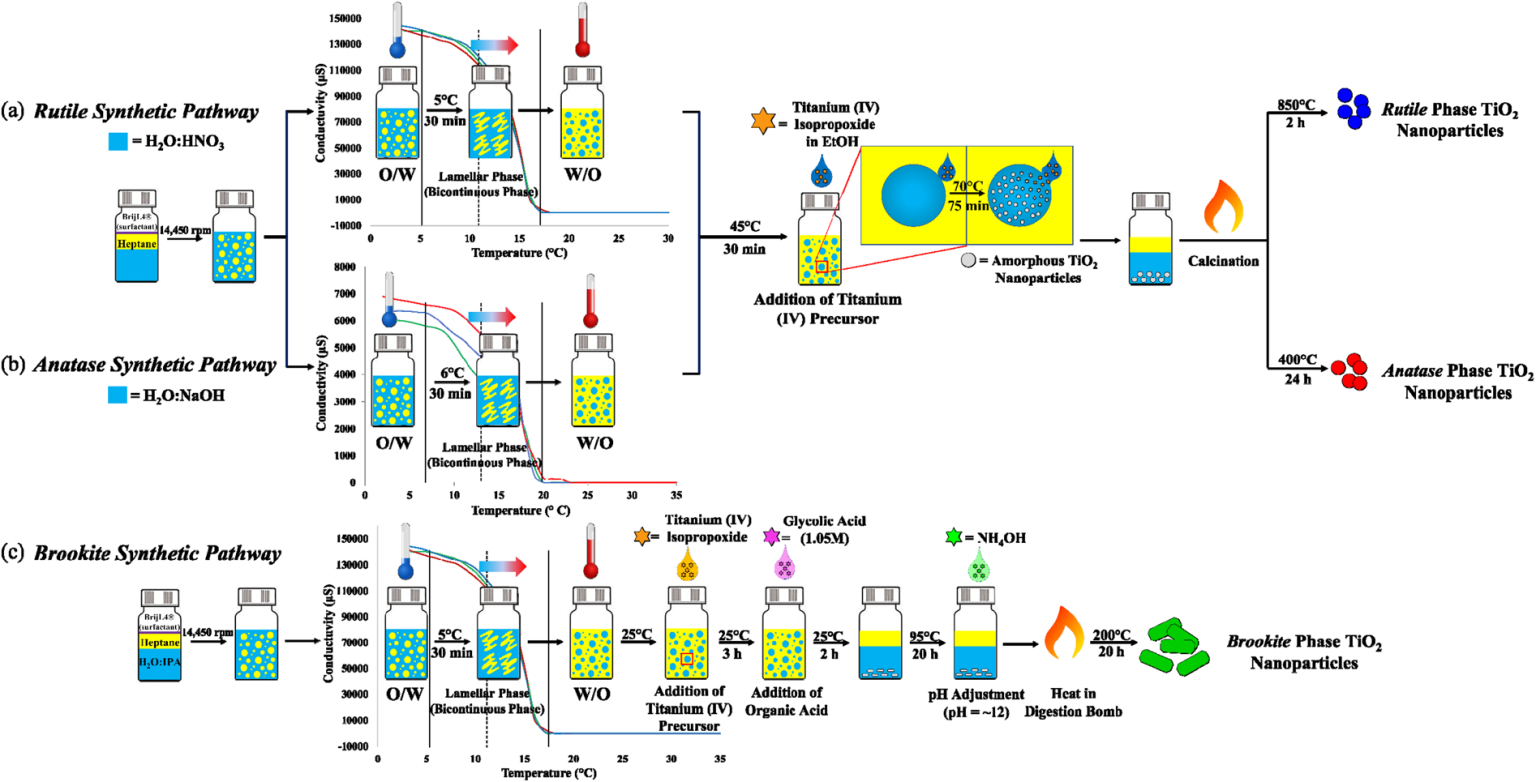
Schematic diagram of the PIT-nano-emulsion synthesis of (a) rutile, (b) anatase, and (c) brookite TiO_2_ highly-pure phase particles. The PIT determination graphs are inserted in each synthetic pathway, showing the inversion of phases at ∼11 °C for moderately and strongly acidic conditions (dashed line, light orange region) and ∼13 °C for alkaline conditions (dashed line, light pink region).

The hydrolysis–condensation reaction that leads the formation of TiO_2_ was adapted to the PIT-nano-emulsion method, employing a multireactor crystallization system (Crystalline™, Technobis, Crystallization Systems, Alkmaar, Netherlands) to obtain high-purity particles of the metal oxide. Three different PIT-nano-emulsion synthesis were designed, each one oriented to isolate one of the respective phases of TiO_2_ (Fig. 1). To reduce the particle size of the amorphous TiO_2_ during the hydrolysis–condensation reaction, the PIT was determined for two types of emulsion systems. The latter varied in terms of the pH of the aqueous phase, which contained either an acidic (HNO_3_, pH ∼0.5) or alkaline (NaOH, pH ∼12) environment. Transformation of amorphous TiO_2_ into the respective TiO_2_ phase depends on the pH and calcination temperature of the suspension.^35–38^ It has been reported that under strong acidic conditions employing nitric acid, the rutile phase formation is favorable due to the slow precipitation rate of the amorphous TiO_2_ during synthesis.^35–37^ In addition, when the amorphous product is calcinated in an acidic environment, it leads to the rutile phase over the other two polymorphs.^35,37^ In contrast, it has been demonstrated that the concentration of NaOH during synthesis plays an important role when stabilizing the anatase phase.^38^ To prevent a polymorphic phase change of anatase to rutile, NaOH acts as an alkalinity precipitant during the hydrolysis–condensation reaction, thus promoting the formation of amorphous sodium titanate. When annealed, the resulting product favors and stabilizes the formation of anatase over rutile.^38^ Therefore, the acidic emulsion system (H_2_O : HNO_3_/heptane, pH ∼0.5) was employed for the synthetic pathway, leading to the rutile phase (Fig. 1a), while the emulsion with alkaline conditions (H_2_O : NaOH/heptane, pH ∼12) was used for the anatase-oriented synthesis (Fig. 1b). After homogenizing the emulsions, conductivity measurements started at 2 °C with the oil-in-water (O/W) system reporting average values of 140 000 μS (H_2_O : HNO_3_/heptane) and 6500 μS (H_2_O : NaOH/heptane) at the starting point. As the emulsion were heated, a phase inversion occurred from O/W (conductive) micro-emulsion to a water-in-oil nano-emulsion (W/O, not conductive). Conductivity measurements dropped once the temperature reached 17 °C (0.030 μS) and 20 °C (0.035 μS) for the strongly acidic and alkaline systems, respectively. As a result, the inversion of phases occurred at ∼11 °C (H_2_O : HNO_3_/heptane) and ∼13 °C (H_2_O : NaOH/heptane). Due to the low pH value reported for the H_2_O : IPA/heptane emulsion system (pH = 4.15), the PIT was achieved by employing the same temperature profile determined for the strongly acidic conditions (Fig. 1c). The H_2_O : IPA/heptane emulsion system was utilized for the synthetic pathway, leading to the isolation of brookite phase nanoparticles.^24^

Once the PIT was identified for each emulsion system, it was used to perform the synthesis of the amorphous TiO_2_ particles. For each synthetic pathway, the respective aqueous phase (H_2_O : HNO_3_, H_2_O : NaOH, or H_2_O : IPA) was entrapped in nanospheres suspended in the oil phase of the emulsion after the inversion of phases. The addition of the titanium(iv) precursor at this point promoted the hydrolysis–condensation reaction to form the amorphous TiO_2_ particles. Continuous high stirring permitted the reaction to occur by the coalescence of the titanium(iv) precursor solution with the aqueous nanospheres within the oil media. In this manner, the nucleation of the amorphous TiO_2_ particles was constrained into the nano-range and mechanically controlled.

### Particle size distribution measurements of amorphous TiO_2_

**Fig. 2:**
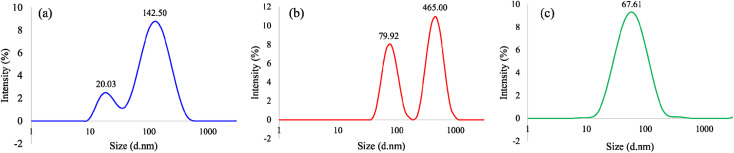
Dynamic light scattering (DLS) spectra of amorphous TiO_2_ particles synthesized employing (a) H_2_O : HNO_3_/heptane, (b) H_2_O : NaOH/heptane, and (c) H_2_O : IPA/heptane emulsion system.

Particle size distribution and polydispersity measurements of the resulting amorphous TiO_2_ before being thermally treated from the bulk (control) and PIT-nano-emulsion syntheses (experimental) were performed. For the control groups, DLS measurements demonstrated average particle diameter distribution values of ∼530, 1600, and 300 d nm for the bulk syntheses employing H_2_O : HNO_3_, H_2_O : NaOH, and H_2_O : IPA, respectively (ESI†). For the PIT-nano-emulsion syntheses products, aliquots from the aqueous supernatant from the emulsion systems with the presumed metal oxide particles were measured after 24 h of being synthesized. The results demonstrated average particle diameter distribution values of 20.03 and 142.50 d nm for particles synthesized employing H_2_O : HNO_3_, 79.92 and 465.00 d nm for particles synthesized in H_2_O : NaOH, and 67.61 d nm for particles employing H_2_O : IPA aqueous phase (Fig. 2). According to these values, syntheses carried out in acidic (H_2_O : HNO_3_/heptane) and alkaline (H_2_O : NaOH/heptane) conditions promoted the formation of particles with a bimodal size distribution, most likely due to the aggregation of the particles over time (Fig. 2a and b). Moreover, the synthesis employing H_2_O : IPA promoted the formation of nanoparticles with a homogeneous (monomodal) size distribution (Fig. 2c). The average polydispersity index (PDI) values obtained were in the range of 0.355–0.486 for each sample. Based on the PDI values, the amorphous TiO_2_ particles measured were moderately monodispersed, which could explain the observed heterogeneity in the size distribution. The results demonstrate that this method produces smaller particles when compared to the bulk syntheses performed as controls (ESI†), possibly by reducing the available volume for the hydrolysis–condensation reaction to occur.

### Thermal treatment of the amorphous TiO_2_ particles

To obtain the desired phase once the amorphous TiO_2_ formed, different approaches to the thermal treatments were undertaken. To control the rutile phase, amorphous TiO_2_ was calcinated at 850 °C for 2 h. Rutile is the most thermodynamically stable phase compared to anatase and brookite, which is favored when treating the amorphous metal oxide at highly elevated temperatures even for short times.^19–22^ In addition, calcination under strong acidic conditions will favor this phase, as discussed in the previous section.^35,37^

To control the production of the highly-pure anatase phase, calcination of amorphous TiO_2_ in the presence of alkaline conditions was conducted at 400 °C for 24 h.^20,22^ It is known that anatase is a metastable phase of TiO_2_, which is susceptible to polymorphic phase transformations, leading to the rutile phase.^19,39^ To avoid the rutile phase, the PIT-nano-emulsion synthesis was carried out employing an aqueous phase containing NaOH in the emulsion system. In addition, calcination of the amorphous TiO_2_ resulting from this synthesis was carried out in the alkaline solution in contrast with the other syntheses, where the products were annealed in acidic environments. As previously mentioned, the role of NaOH during the synthesis and calcination of the metal oxide is to stabilize the anatase phase and promote its formation.^38^

In terms of brookite, the PIT-nano-emulsion synthesis and thermal treatment leading to this phase were distinct to the synthetic pathways designed for the rutile and anatase phases. Two experimental steps were included once the amorphous TiO_2_ formed after the PIT. The addition of polymorph-regulating agents such as glycolic acid and NH_4_OH was coupled to the PIT-nano-emulsion synthesis to promote the formation of a titanium glycolate complex, as reported by Mamakhel *et al.*^24^ An advantage of introducing the PIT-nano-emulsion method is the formation of the complex inside the aqueous nanospheres to control its particle size. Once formed, the amorphous TiO_2_ underwent thermal treatment at 200 °C inside a digestion bomb for 20 h.^24^ Combined, these experimental steps in the designed PIT-nano-emulsion synthesis after thermal treatment could lead to the formation of highly-pure brookite phase nanoparticles employing fewer toxic compounds, reducing the quantity of starting materials, and with minimum energy input when compared to previously employed methods (ESI†).^24–26^

### Raman spectroscopy

**Fig. 3:**
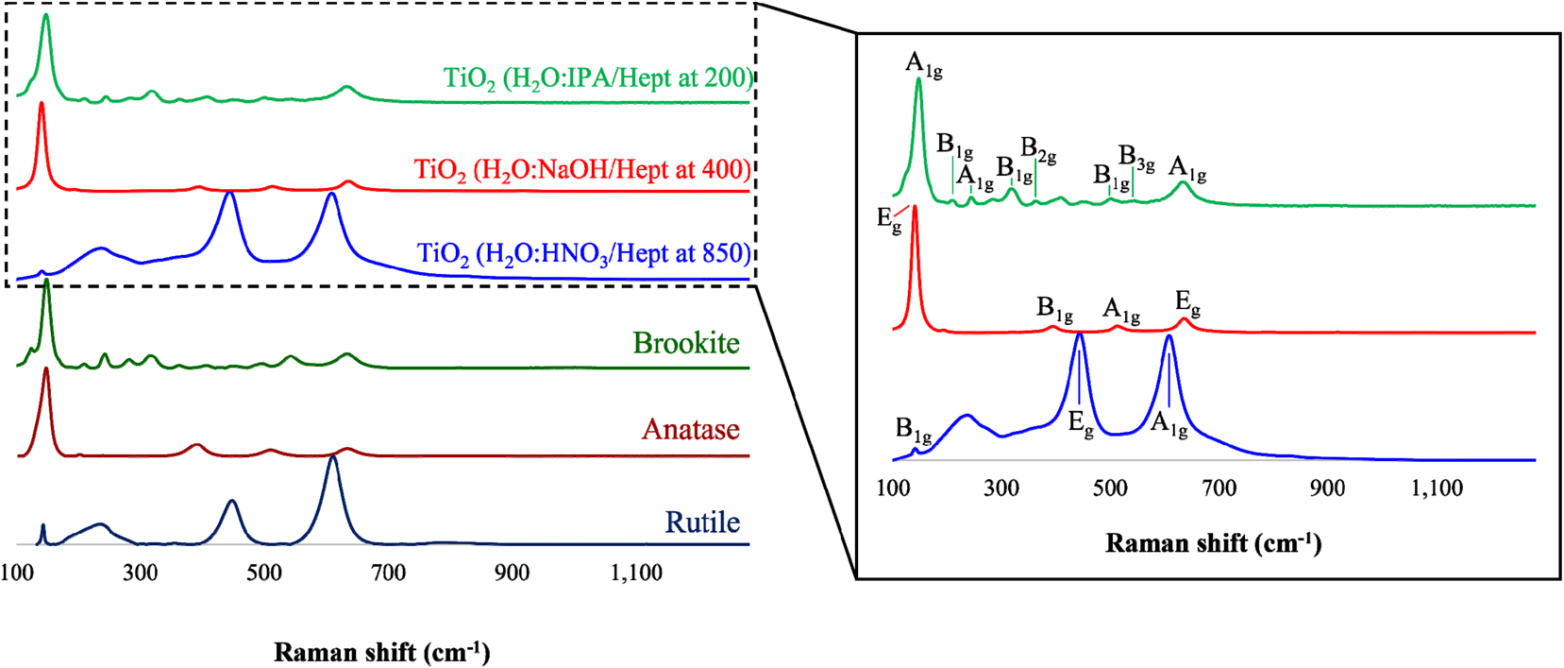
(Left) Raman spectra overlay of simulated rutile (navy blue), anatase (dark red), and brookite (dark green) phases, as well as the Raman spectra of the TiO_2_ particles synthesized through the PIT-nano-emulsion method, employing H_2_O : HNO_3_/heptane (blue), H_2_O : NaOH/heptane (red), and H_2_O : IPA/heptane (green) emulsion systems and after thermal treatment. (Right) Raman spectra for TiO_2_ particles under each condition with the prominent Raman modes identified.

To corroborate that the resulting product from the PIT-nano-emulsion syntheses were amorphous, Raman spectroscopy was performed on the samples before thermal treatment. The results demonstrate that the TiO_2_ particles were amorphous due to the absence of characteristic Raman modes from each crystalline phase of the metal oxide, as well as the presence of high background in the spectra (ESI†). The representative Raman spectra of the calcinated or thermally-treated TiO_2_ particles were collected in the range from 1300 to 100 cm^−1^ (Fig. 3). This analysis initially confirmed that the resulting particles were synthesized as highly-pure phases of TiO_2_ with the presence and absence of different characteristic Raman shifts when compared to the simulated spectra of each phase from the literature. The particles synthesized employing the acidic emulsion system (H_2_O : HNO_3_/heptane) and calcinated at 850 °C presented the three distinctive Raman shifts, the B_1g_ (148 cm^−1^), E_g_ (446 cm^−1^), and A_1g_ (603 cm^−1^) modes, that are characteristic of the rutile phase.^24,40–42^ Moreover, the Raman spectra of the particles previously formed in alkaline conditions (H_2_O : NaOH/heptane) and calcinated at 400 °C presented only anatase characteristic Raman shifts. The intense (139 cm^−1^) and weak (646 cm^−1^) Raman signals observed correspond to the E_g_ mode of anatase, while the other signals at 384 and 502 cm^−1^ account for the A_1g_ and B_1g_ modes of this phase, respectively.^24,35,40–42^ The Raman spectra of the nanoparticles obtained employing the H_2_O : IPA/heptane emulsion system and thermal treatment at 200 °C presented several Raman signals distinctive of the brookite phase. These signals correspond to the A_1g_ (149, 249, and 641 cm^−1^), B_1g_ (210, 323, and 504 cm^−1^), B_2g_ (348 cm^−1^), and B_3g_ (546 cm^−1^) modes characteristic of the brookite phase and are in accordance with the literature.^24^ In particular, the presence of a Raman shift at 323 cm^−1^ (B_1g_) usually denotes the presence of highly-pure brookite phase. The absence of characteristic signals of other TiO_2_ phases in the spectra of each product demonstrates that the resulting particles were obtained as single-phases.

### Powder X-ray diffraction (PXRD)

Control syntheses (bulk syntheses) were performed without employing the PIT-nano-emulsion method but under the same pH and thermal conditions. After the syntheses, PXRD analysis confirmed that the products were amorphous due to the absence of Bragg reflections (ESI†). Moreover, calcinated products from bulk syntheses employing strongly acidic and alkaline conditions resulted in pure phases. Nevertheless, the product thermally treated and obtained under moderately acidic conditions resulted in a mixture of phases, presenting phase impurities based on the presence of distinctive signals from other polymorphs in the diffractogram (ESI†).

**Fig. 4:**
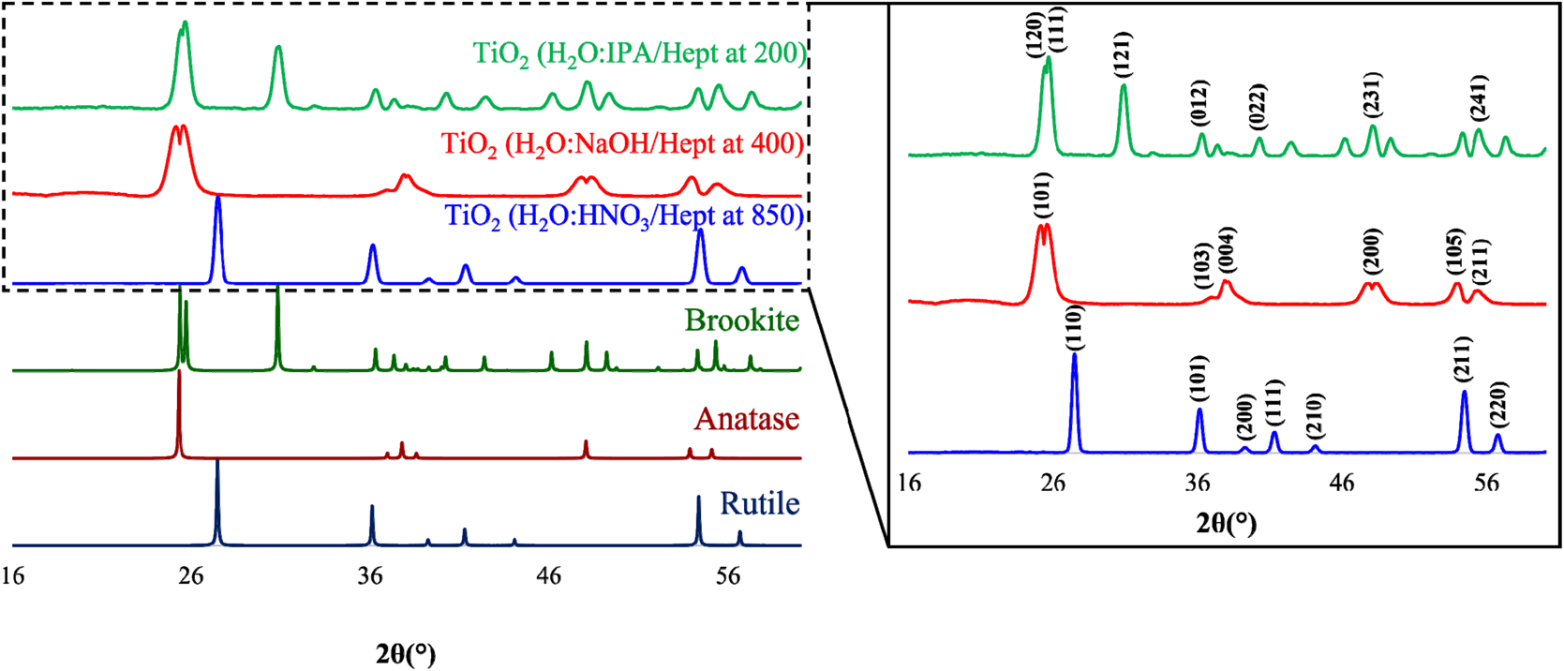
(Left) PXRD overlay of simulated rutile (ICSD 165920, navy blue),^43^ anatase (ICSD 154601, dark red),^44^ and brookite (ICSD 154605, dark green)^44^ phases, with the diffractograms of the TiO_2_ particles synthesized through the PIT-nano-emulsion method employing H_2_O : HNO_3_/heptane (blue), H_2_O : NaOH/heptane (red), and H_2_O : IPA/heptane (green) emulsion systems and thermal treatment. (Right) Inset of the PXRD overlay for the experimentally obtained TiO_2_ particles showing indexed Bragg reflections.

The representative PXRD diffractograms of the TiO_2_ particles calcinated or thermally treated after their respective PIT-nano-emulsion synthesis are shown in Fig. 4. Each diffractogram reveals a high degree of crystallinity for the isolated particles due to the low amorphous background and relatively strong Bragg peaks observed compared to the amorphous TiO_2_ obtained after synthesis before the thermal treatment (ESI†). This analysis unambiguously confirmed that the resulting particles were obtained as highly-pure phases of TiO_2_ based on the presence of characteristic reflections for each TiO_2_ polymorph. The peaks observed in the powder pattern of the particles synthesized employing the H_2_O : HNO_3_/heptane emulsion system are characteristic of the rutile phase. These signals can be observed at 27.45, 36.11, 39.37, 41.27, 44.11, 54.39, and 56.97° in 2*θ* and can be indexed to the (110), (101), (200), (111), (210), (211), and (220) planes corresponding to the pure rutile phase.^24,40–42^ Moreover, the crystal phase of the particles synthesized through the H_2_O : NaOH/heptane emulsion system was confirmed as the highly-pure anatase phase. The characteristic peaks observed at 2*θ* = 25.86, 37.20, 37.98, 48.17, 54.19, and 55.46° correspond to the planes (101), (103), (004), (200), (105), and (211) observed for pure anatase.^24,40–42^ Furthermore, highly-pure phase brookite nanoparticles were confirmed as the resulting product from the synthesis employing the H_2_O : IPA/heptane emulsion system and thermal treatment. The presence of the characteristic indexed Bragg peaks at 25.51 (120), 25.79 (111), 30.98 (121), 36.44 (012), 40.42 (022), 48.29 (231), and 55.63° (241) in 2*θ* confirmed the isolation of brookite as the pure phase.^24,41^ In each diffractogram of the products analyzed, no other peaks corresponding to additional phase impurities were observed. These results, along with Raman spectra analysis, suggest that both the chemical nature of the emulsion system (*e.g.*, pH) and the specific thermal treatment (temperature and time) employed influences the selective isolation of the respective titania polymorph. The following control experiments were also performed to support these observations. At a calcination temperature of 200 °C, the suspension (amorphous titania + aqueous phase) composed of H_2_O : IPA (pH ∼4.15) yields highly-pure brookite, whereas, if suspended in H_2_O : NaOH (pH ∼12), highly-pure anatase phase is observed by PXRD (ESI†). Moreover, at a calcination temperature of 400 °C, the suspension (amorphous titania + aqueous phase) composed of H_2_O : HNO_3_ (pH ∼0.5) yields a mixture of anatase-rutile, whereas if suspended in H_2_O : NaOH (pH ∼12), highly-pure anatase phase is isolated, as demonstrated by PXRD (ESI†). Therefore, it is confirmed that the designed PIT-nano-emulsion syntheses presented in this work lead to the formation of phase-selective TiO_2_ particles.

### Scanning electron microscopy coupled with energy dispersive spectroscopy (SEM-EDS)

**Fig. 5:**
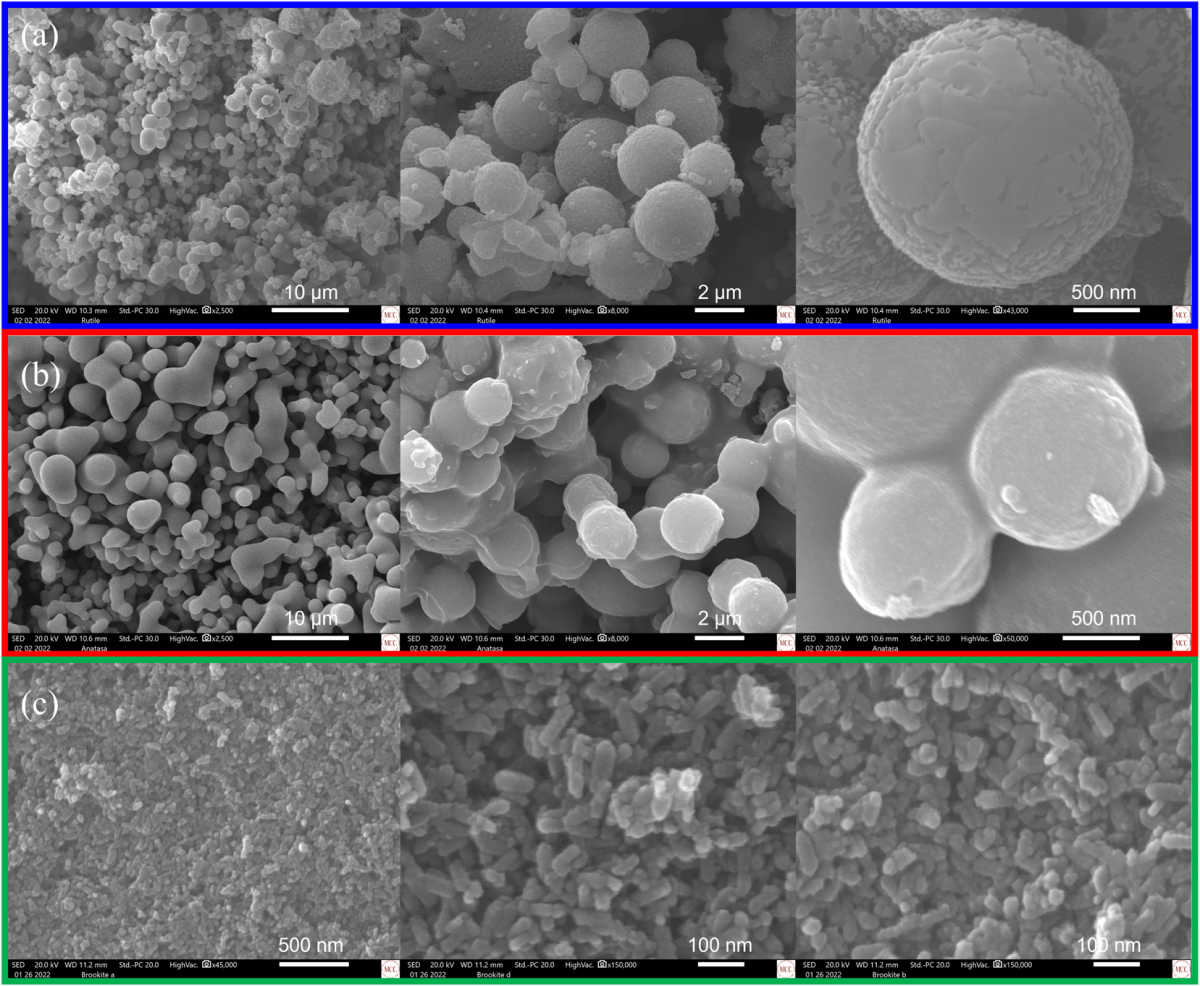
Scanning electron micrographs of highly-pure (a) rutile, (b) anatase, and (c) brookite phase TiO_2_ after being synthesized through the PIT-nano-emulsion method and thermal treatment.

Morphological studies through SEM-EDS were performed on the three highly-pure TiO_2_ phases particles obtained. The representative SEM images collected show particles with well-defined morphologies (Fig. 5). In Fig. 5a, it can be observed that the rutile phase particles present a uniform spherical morphology with varied size diameters (30–800 nm, average ∼350 nm). In some instances, the agglomeration of the spheres can be observed. For the anatase phase particles, the morphology observed was mostly irregular-spherical (Fig. 5b). However, high agglomeration of these non-uniform spheres was observed, resulting in clusters. The resulting diameter for these particles was in the range of 20–500 nm (average ∼450 nm). Moreover, SEM analysis of the brookite-phase nanoparticles demonstrated high agglomeration and cluster formation. Nevertheless, these brookite phase nanoparticles presented higher morphological uniformity among the samples analyzed. Fig. 5c depicts a rodlike morphology for these nanoparticles with an average size diameter of ∼75 nm. The observed particle sizes are comparable to those determined by DLS (ESI†). Nevertheless, when the crystallite size of each of the TiO_2_ phase obtained was approximated using PXRD and the Debye–Scherrer equation, it is found that the crystallite size is much smaller than the particle size determined by DLS and SEM-EDS (ESI†). The observed discrepancy in particle *vs.* crystalline size might indicate that TiO_2_ particles are polycrystalline and composed of agglomerated TiO_2_ nanoparticles.

**Fig. 6:**
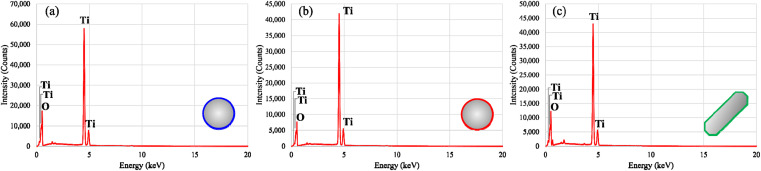
Energy dispersive spectra of highly-pure (a) rutile, (b) anatase, and (c) brookite TiO_2_ particles after being synthesized through the PIT-nano-emulsion syntheses and thermal treatment. EDS spectra includes the insertion of the morphology observed for each respective particle (sphere or rod-like shaped) in the scanning electron micrographs.

The representative EDS spectra of these materials present the characteristic signals of titanium (Ti) and oxygen (O), which are present in the molecular structure of the metal oxide (Fig. 6). This corroborates the elemental composition of the products as the particles are only comprised of TiO_2_. The absence of other element signals suggests the high chemical purity content of TiO_2_, without the presence of trace amounts of starting materials from the PIT-nano-emulsion syntheses (*e.g.*, titanium(iv) isopropoxide, Brij L4®, heptane, HNO_3_, NaOH, glycolic acid, and NH_4_OH) or the thermal treatment process.

## Conclusions

Herein, we reported for the first time three different PIT-nano-emulsion syntheses and calcination treatments leading to the selective formation of multiple and highly-pure phases of TiO_2_ as particles. The application of the low-energy emulsification method known as the PIT in the multireactor crystallization system (Crystalline™, Technobis, Crystallization Systems, Alkmaar, Netherlands), limited the TiO_2_ hydrolysis–condensation reaction space available in the nano-range. The designed PIT-nano-emulsion syntheses were specifically oriented to isolate rutile, anatase, and brookite phase TiO_2_ particles using different emulsion systems (*i.e.*, H_2_O : HNO_3_/heptane, H_2_O : NaOH/heptane, and H_2_O : IPA/heptane; respectively). Tailored calcination temperatures for each polymorph (850 °C for 2 h for rutile, 400 °C for 24 h for anatase, and 200 °C for 20 h for brookite) aided the form selection. It was demonstrated that pH and thermal treatment contribute to the isolation of these three phases of TiO_2_ as highly-pure phases. From the three reported PIT-nano-emulsion syntheses, particle size values below <465 d nm were obtained for amorphous TiO_2_ particles. Solid-state characterization through Raman spectroscopy and PXRD confirmed that after thermal treatment, the TiO_2_ particles were comprised of a single phase and thus had high purity. SEM analysis provided evidence of the well-defined morphologies observed in these particles. Here, rutile and anatase particles are spherical shaped, while brookite nanoparticles present a rodlike shape. These results demonstrate the capacity of the reported PIT-nano-emulsion syntheses, along with the respective thermal treatment employed, to control not only the particle size but also the phase purity of the produced TiO_2_ particles. The latter was achieved employing fewer toxic compounds, reducing the quantity of starting materials, and with minimal energy input, particularly for the almost elusive brookite phase. Therefore, the designed synthetic pathways could provide an alternative approach to obtain nanoparticles for studies were the purity of the TiO_2_ phase is critical.

## Author contributions

Gabriel Quiñones Vélez: conceptualization, data curation, formal analysis, investigation, methodology, validation, visualization, writing-original draft, writing-review & editing. Diego Soto Nieves: data curation, formal analysis, investigation, methodology. Anushka Castro Vázquez: data curation, formal analysis, investigation, methodology. Vilmalí López-Mejías: conceptualization, funding acquisition, investigation, methodology, project administration, resources, supervision, validation, visualization, writing–review & editing.

## Conflicts of interest

There are no conflicts of interest to declare.

## Supplementary Material

NA-005-D2NA00390B-s001
