# Supplementary material for: Polymorphic control in titanium dioxide particles
Source: Nanoscale Adv. 2022 Nov 23;5(2):425–34. doi: 10.1039/d2na00390b (PMC9846715; doi:10.1039/d2na00390b)
Supplement: NA-005-D2NA00390B-s001 [file NA-005-D2NA00390B-s001.pdf]

Electronic Supplementary Material (ESI) for Nanoscale Advances.
This journal is © The Royal Society of Chemistry 2022

Supporting Information

Polymorphic Control in Titanium Dioxide Particles

Gabriel Quiñones Vélez[a,b], Diego Soto Nieves[a,b], Anushka Castro Vázquez[b,c] and Vilmalí López-Mejías[a,b,]*

[a] Department of Chemistry, University of Puerto Rico, Río Piedras
San Juan, Puerto Rico, 00931 United States.
[b] Crystallization Design Institute, Molecular Sciences Research Center,
University of Puerto Rico, San Juan, Puerto Rico, 00926 United States.
[c] Department of Accounting, University of Puerto Rico, Río Piedras
San Juan, Puerto Rico, 00931 United States.
*vilmali.lopez@upr.edu

Table of content.

S 1. Bulk Syntheses of TiO_2 particles
- S 1.1. Syntheses
- S 1.2. Particle Size Distribution of Bulk Material
- S 1.3. Micro-powder X-ray Diffraction of Bulk Material

S 2. Phase Inversion Temperature (PIT) Determination
S 3. Particle Size Distribution of Amorphous TiO_2 Particles
S 4. Raman Vibrational Spectroscopy
S 5. Micro-powder X-ray Diffraction (PXRD)
S 6. Comparison of Experimental Conditions for TiO_2 Brookite Syntheses
S 7. Size Comparison of the Synthesized TiO_2 Particles by Different Methods
S 8. References

1. Bulk Syntheses of TiO_2 Particles

1.1. Syntheses.

Bulk Synthesis of Rutile Phase TiO_2

A mixture of H_2O:HNO_3 was prepared separately by diluting 6 mL of nitric acid in 100 mL of nanopure water. In a glass container, 50mL of the H_2O:HNO_3 solution were added and heated in a hot plate at 35°C for 15 min. In another container, the titanium (IV) precursor solution was prepared by mixing 15 mL of ethanol, 5 mL of glacial acetic acid and 5 mL of titanium (IV) isopropoxide. The titanium (IV) precursor solution was added to the H_2O:HNO_3 mixture and stirred at 350 rpm. Here, the glass container was sealed with aluminum foil to prevent evaporation. The resulting mixture was heated at 70°C for 75 min.

Bulk Synthesis of Anatase Phase TiO_2

A mixture of H_2O:NaOH was prepared separately by diluting 6 mL of NaOH 1.0 M in 100 mL of nanopure water. In a glass container, 50mL of the H_2O:NaOH solution were added and heated in a hot plate at 35°C for 15 min. In a separate container, the titanium (IV) precursor solution was prepared by mixing 15 mL of ethanol, 5 mL of glacial acetic acid and 5 mL of titanium (IV) isopropoxide. The titanium (IV) precursor solution was added to the H_2O:NaOH mixture and stirred at 350 rpm. Here, the glass container was sealed with aluminum foil to prevent evaporation. The resulting mixture was heated at 70°C for 75 min.

Bulk Synthesis of Brookite Phase TiO_2

For the brookite phase TiO_2 bulk synthesis as control, the experimental procedure reported by Mamakhel *et al.* was followed.[1]

1.2. Particle Size Determination of the Bulk Material.

Samples were prepared by taking 50 μL aliquots of the resultant suspension presumed to contain the bulk amorphous TiO_2. They were transferred in disposable polystyrene cuvettes (REF: 67.754, 10 x 10 x 45 mm, Sarsted, Germany) and diluted with nanopure water in a 1:40 ratio. The cuvettes containing the samples remained undisturbed near the Zetasizer for 30 min prior to the measurements. Afterwards, size measurements were performed after 2 min of sample equilibration inside the instrument at room temperature (25°C).

Tables S1.2.1-S1.2.3 summarize the DLS parameters and values for the three different bulk syntheses products in nanopure water. Figures S1.2.1-S1.2.3 depict the DLS spectra showing the particle size distribution of amorphous TiO_2 particles from the three respective bulk syntheses.

Table S1.2.1. Dynamic light scattering parameters and values after analyzing the product from the bulk synthesis employing H_2O:HNO_3.

Bulk Synthesis employing H_2O:HNO_3				
Run	Size (d.nm)	% Intensity	St Dev (d.nm)	PDI
1	40.40	75.7	17.33	0.468
	681.9	24.3	370.4	
	0.000	0.0	0.000	
2	38.78	74.3	16.25	0.485
	521.4	21.6	233.2	
	4533	4.1	870.3	
3	41.89	79.1	22.09	0.470
	408.1	16.6	178.8	
	4667	4.3	804.0	
Average	40.39	76.4	18.84	0.474
	553.9	20.8	305.0	
	4579	2.8	865.5	

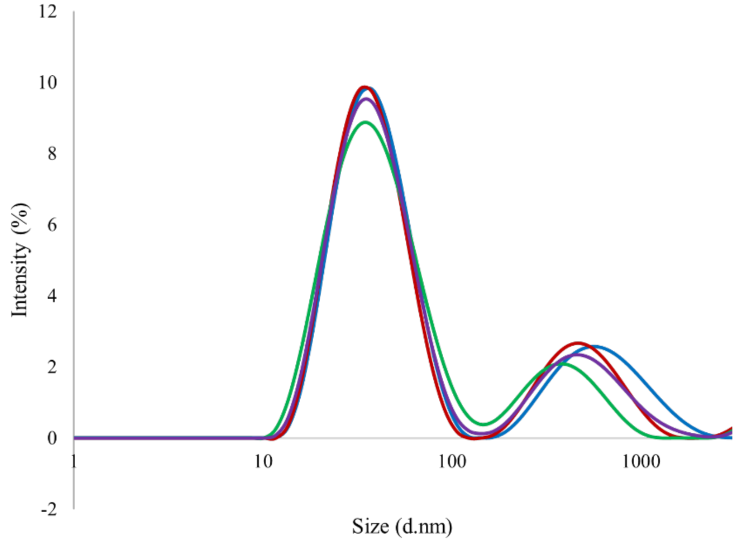

Figure S1.2.1. DLS spectra showing particle size distribution for each run (red, blue and green) and average size values of 40.39 and 533.9 d.nm (purple).

Table S1.2.2. Dynamic light scattering parameters and values after analyzing the product from the bulk synthesis employing H_2O:NaOH

Bulk Synthesis employing H_2O:NaOH				
Run	Size (d.nm)	% Intensity	St Dev (d.nm)	PDI
1	1444	100.0	170.8	0.536
	0.000	0.0	0.000	
	0.000	0.0	0.000	
2	1984	100.0	308.2	0.393
	0.000	0.0	0.000	
	0.000	0.0	0.000	
3	1470	100.0	156.9	0.581
	0.000	0.0	0.000	
	0.000	0.0	0.000	
Average	1633	100.0	333.9	0.503
	0.000	0.0	0.000	
	0.000	0.0	0.000	

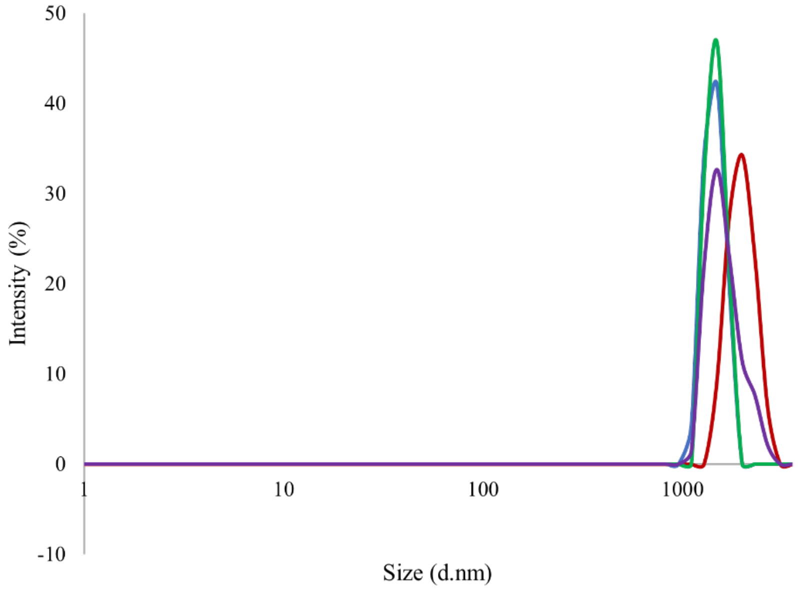

Figure S1.2.2. DLS spectra showing particle size distribution (d. nm) for each run (red, blue and green) and average size values of 1633 d.nm (purple).

Table S1.2.3. Dynamic light scattering parameters and values after analyzing the product from the bulk synthesis employing H_2O:IPA

Bulk Synthesis employing H_2O:IPA				
Run	Size (d.nm)	% Intensity	St Dev (d.nm)	PDI
1	42.99	78.0	28.03	0.530
	297.9	17.1	121.8	
	4965	4.9	629.3	
2	45.17	84.9	29.98	0.468
	355.6	11.0	151.5	
	4663	4.2	805.5	
3	40.21	80.2	23.17	0.377
	277.2	16.5	122.3	
	4880	3.3	687.3	
Average	42.92	81.1	26.92	0.458
	305.9	14.8	132.5	
	4841	4.1	720.3	

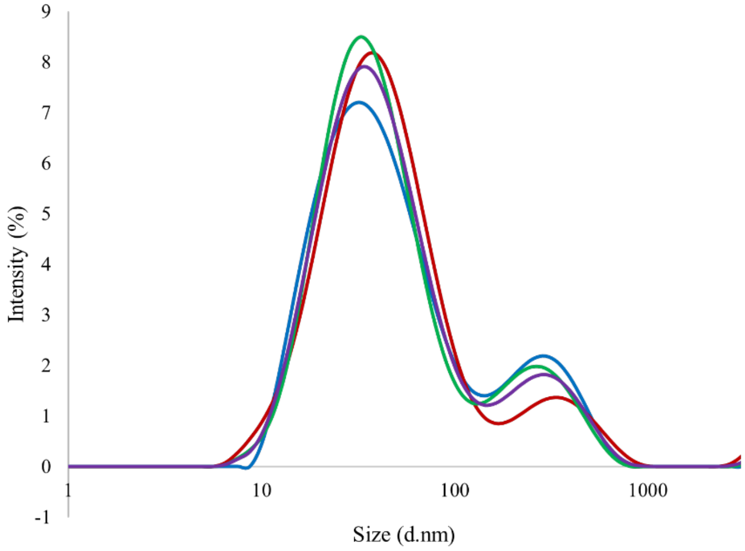

Figure S1.2.3. DLS spectra showing particle size distribution (d. nm) for each run (red, blue and green) and an average size value of 42.92 and 305.9 d.nm (purple).

1.3. Micro-powder X-ray Diffraction of Bulk Material.

Figures S1.3.1 depict an overlay of the experimental powder pattern of the TiO_2 particles obtained through the bulk syntheses without thermal treatment, with the spectra of the three standards rutile (ICSD 165920)[2], anatase (ICSD 154601)[3], and brookite (ICSD 154605)[3]. Figure S1.3.2 depict an overlay of the experimental powder pattern of the TiO_2 particles obtained through the bulk syntheses and thermally treated with the respective standards.

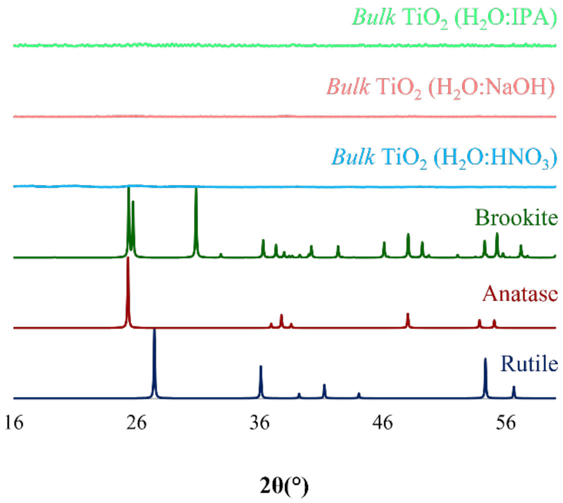

Figure 1.3.1. PXRD overlay of simulated rutile (ICSD 165920, navy blue)[2], anatase (ICSD 154601, dark red)[3], and brookite (ICSD 154605, dark green)[3] phases, with the diffractograms of the TiO_2 particles synthesized through the bulk syntheses without thermal treatment, employing $H_2O:HNO_3$ (light blue), H_2O:NaOH (light red), and H_2O:IPA (light green) conditions.

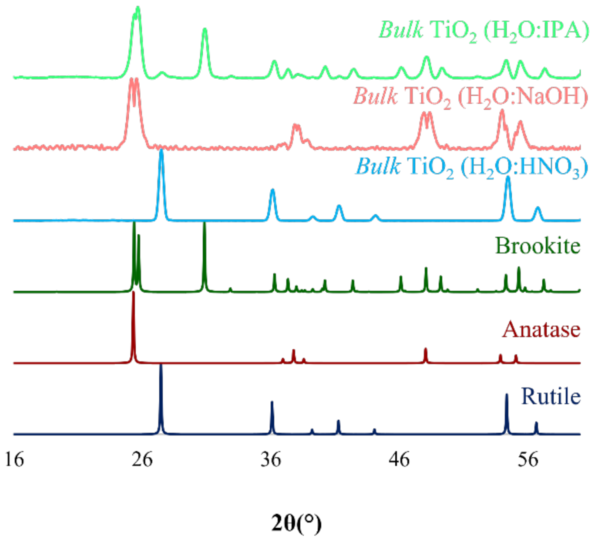

Figure 1.3.1. PXRD overlay of simulated rutile (ICSD 165920, navy blue)[2], anatase (ICSD 154601, dark red)[3], and brookite (ICSD 154605, dark green)[3] phases, with the diffractograms of the TiO_2 particles synthesized through the bulk syntheses after thermal treatment, employing $H_2O:HNO_3$ (light blue), H_2O:NaOH (light red), and H_2O:IPA (light green) conditions.

2. Phase Inversion Temperature (PIT) Determination

For the H_2O:HNO_3/Heptane and H_2O:NaOH/Heptane emulsion systems, the PIT was determined as follows. The respective mixture (respective aqueous phase, heptane and BrijL4®) was homogenized using an IKA T10 Basic Ultra Turrax (IKA Works Inc., Wilmington, NC), for 30 sec at a speed of “4” (14,450 rpm equivalent). The vial was situated in a jacketed beaker, with a 20.3 cm (8”) stainless steel RTD temperature probe (VWR®, VWR International). The conductivity of the emulsion was measured with a Fisherbrand Accumet BasicAB30 conductivity meter (Fisher Scientific UK, Loughborough, UK). The bath temperature was controlled with a Julabo F32-ME Refrigerated/Heating Circulator (JULABO GmbH, Seelbach, Germany). Both the vial and the bath contained magnetic stir bars stirring at 300 rpm using a VWR® Professional Hot Plate Stirrer (97042-714, VWR®, VWR International). The temperature of the emulsion was allowed to reach 2°C in the bath before starting the measurements. The temperature profile started at 2°C and ended at 37°C at a heating rate of 1°C/min. The conductivity of the respective mixture was recorded in 1-degree intervals. Figure S2.1-2.2 depicts the PIT determination curves for the H_2O:HNO_3/Heptane and H_2O:NaOH/Heptane emulsion systems, respectively.

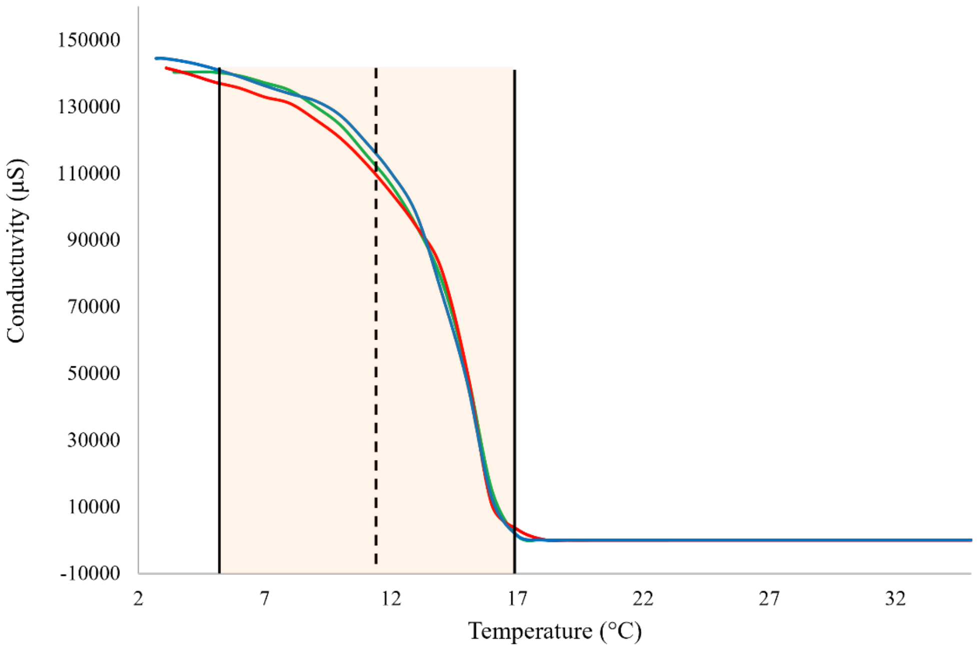

Figure S2.1. Nano-emulsion PIT determination of the H_2O:HNO_3/Heptane emulsion system, showing the phase inversion occurs at approximately ~ 11°C (dashed line). Depicted by the light orange region is the range for the phase inversion which starts at 5°C and ends at 17°C.

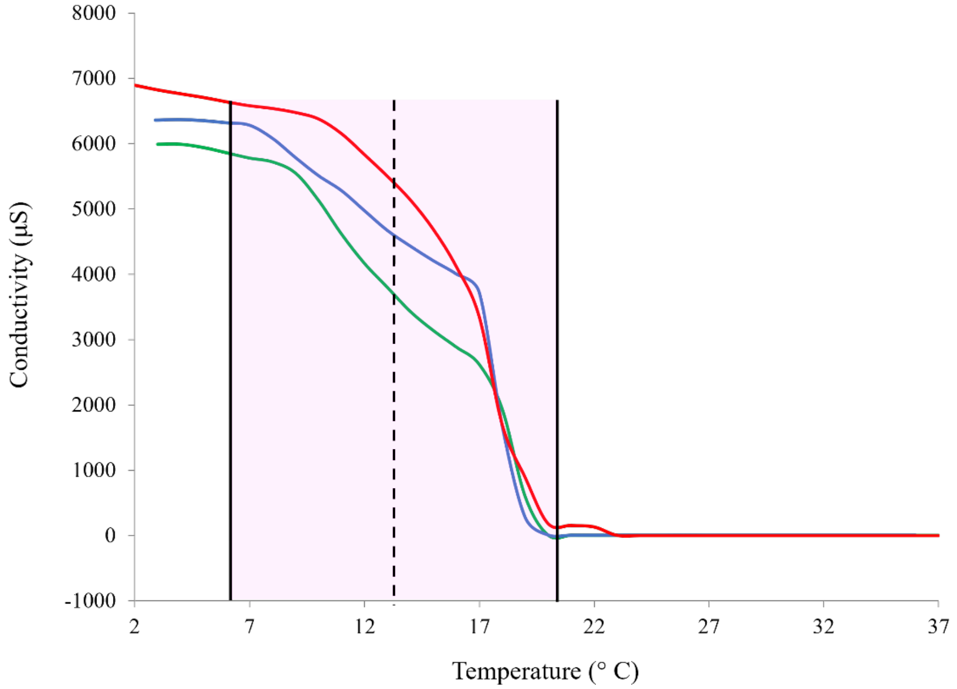

Figure S2.2. Nano-emulsion PIT determination of the H_2O:NaOH/Heptane emulsion system, showing the phase inversion occurs at approximately ~ 13°C (dashed line). Depicted by the light pink region is the range for the phase inversion which starts at 6°C and ends at 20°C.

3. Particle Size Distribution of Amorphous TiO_2 Particles

Samples were prepared by taking 50 μL aliquots of the supernatant from the respective PIT-nano-emulsion synthesis aqueous phase. They were transferred in disposable polystyrene cuvettes (REF: 67.754, 10 x 10 x 45 mm, Sarsted, Germany) and diluted with nanopure water in a 1:40 ratio. The cuvettes containing the samples remained undisturbed near the Zetasizer for 30 min prior to the measurements. Afterwards, size measurements were performed after 2 min of sample equilibration inside the instrument at room temperature (25°C).

Tables S3.1-S3.3 summarize the DLS parameters and values for the three different PIT-nano-emulsion syntheses products in nanopure water. Figures S3.1-S3.3 depict the DLS spectra showing the particle size distribution of amorphous TiO_2 nanoparticles from the three respective PIT-nano-emulsion syntheses.

Table S3.1. Dynamic light scattering parameters and values after analyzing the PIT-nano-emulsion synthesis product employing the H_2O:HNO_3/Heptane emulsion system.

**Emulsion System: H_2O:HNO_3/Heptane**				
Run	Size (d.nm)	% Intensity	St Dev (d.nm)	PDI
1	138.3	82.6	70.81	0.489
	21.78	16.2	7.538	
	4795	1.2	726.5	
2	140.3	86.3	74.65	0.476
	18.73	13.7	5.479	
	0.000	0.0	0.000	
3	152.6	83.4	85.43	0.493
	22.41	16.6	7.844	
	0.000	0.0	0.000	
Average	142.5	85.0	77.93	0.486
	20.03	14.6	6.103	
	4795	0.4	726.5	

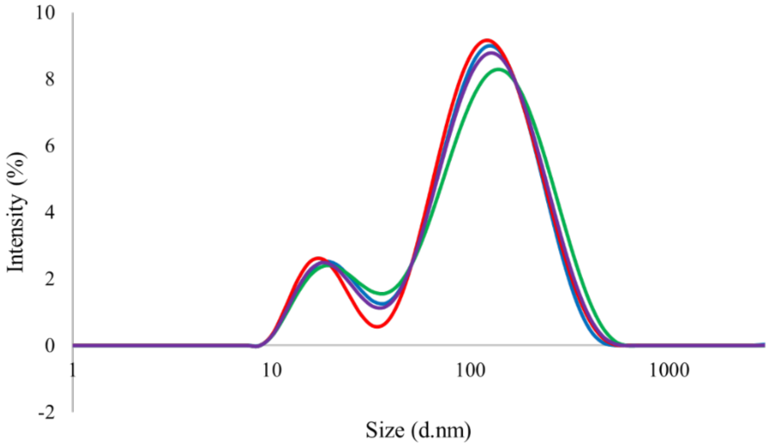

Figure S3.1. DLS spectra showing particle size distribution for each run (red, blue and green) and average size values of 142.5 and 20.03 d.nm (purple).

Table S3.2. Dynamic light scattering parameters and values after analyzing the PIT-nano-emulsion synthesis product employing the H_2O:NaOH/Heptane emulsion system.

**Emulsion System: H_2O:NaOH/Heptane**				
Run	Size (d.nm)	% Intensity	St Dev (d.nm)	PDI
1	431.6	61.2	121.9	0.571
	73.34	37.5	18.89	
	5560	1.3	0.000	
2	445.8	57.5	111.4	0.348
	76.88	42.5	18.21	
	0.000	0.0	0.000	
3	520.0	57.3	159.5	0.539
	88.95	41.6	26.28	
	5560	1.1	0.000	
Average	465.0	58.7	137.9	0.486
	79.92	40.5	22.51	
	5560	0.8	6.104e-5	

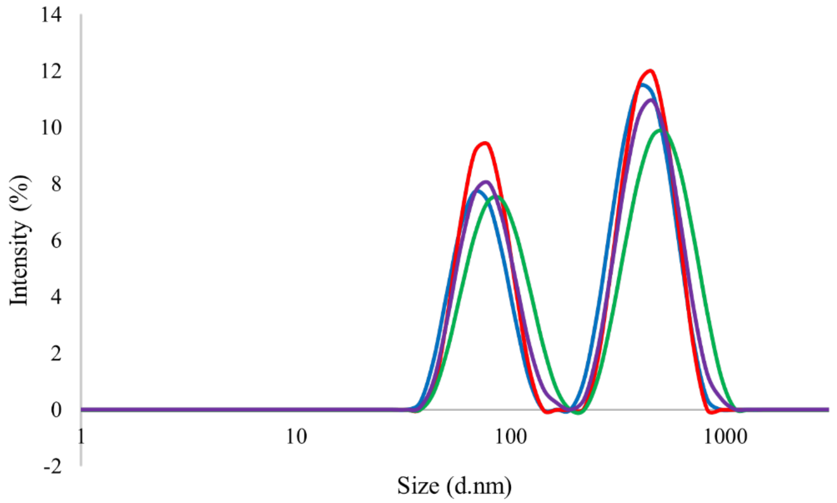

Figure S3.2. DLS spectra showing particle size distribution (d. nm) for each run (red, blue and green) and average size values of 465.0 and 79.92 d.nm (purple).

Table S3.3. Dynamic light scattering parameters and values after analyzing the PIT-nano-emulsion synthesis product employing the H_2O:IPA/Heptane emulsion system.

**Emulsion System: H_2O:IPA/Heptane**				
Run	Size (d.nm)	% Intensity	St Dev (d.nm)	PDI
1	66.95	93.0	39.34	0.385
	4687	7.0	791.7	
	0.000	0.0	0.000	
2	70.28	95.5	53.94	0.344
	5225	4.1	459.9	
	8.461	0.4	1.329	
3	65.86	97.6	35.84	0.336
	5357	2.4	340.3	
	0.000	0.0	0.000	
Average	67.61	95.5	43.80	0.355
	4971	4.5	704.7	
	0.000	0.0	0.000	

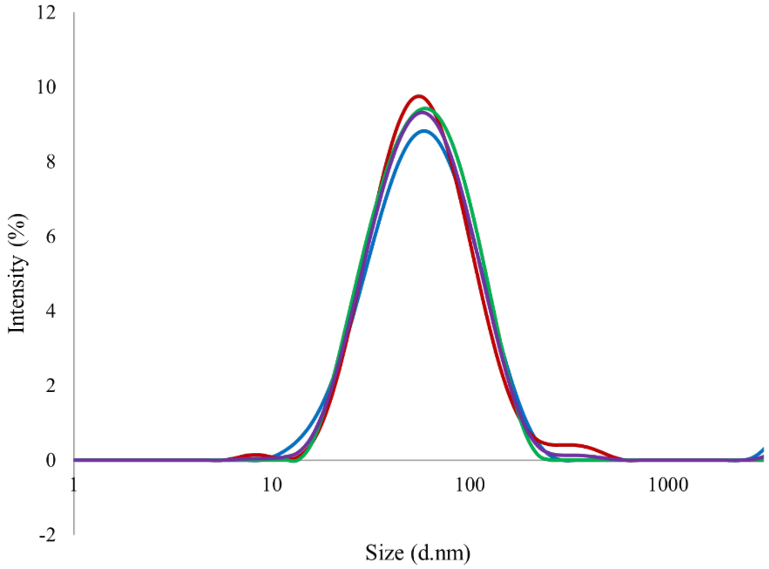

Figure S3.3. DLS spectra showing particle size distribution (d. nm) for each run (red, blue and green) and an average size value of 67.61 d.nm (purple).

4. Raman Vibration Spectroscopy

Figures S4.1-S4.3 depict an overlay of the experimental Raman spectra of the TiO_2 particles obtained through the PIT-nano-emulsion synthesis and thermally treated, with the spectra of the three standards (rutile, anatase and brookite). Figure S4.4 depict an overlay of the experimental Raman spectra of the TiO_2 particles obtained through the PIT-nano-emulsion method without being thermally treated.

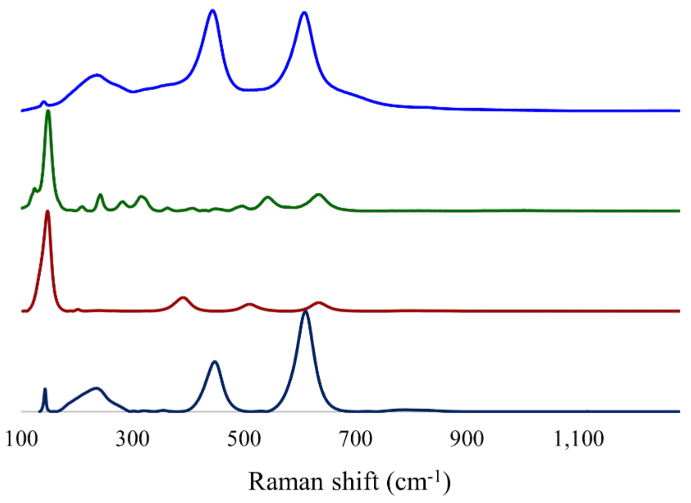

Figure S4.1. Raman spectra overlay of rutile (navy blue), anatase (dark red), and brookite (dark green) standards, and the TiO_2 particles employing the H_2O:HNO_3/Heptane system and calcinated at 850°C (blue).

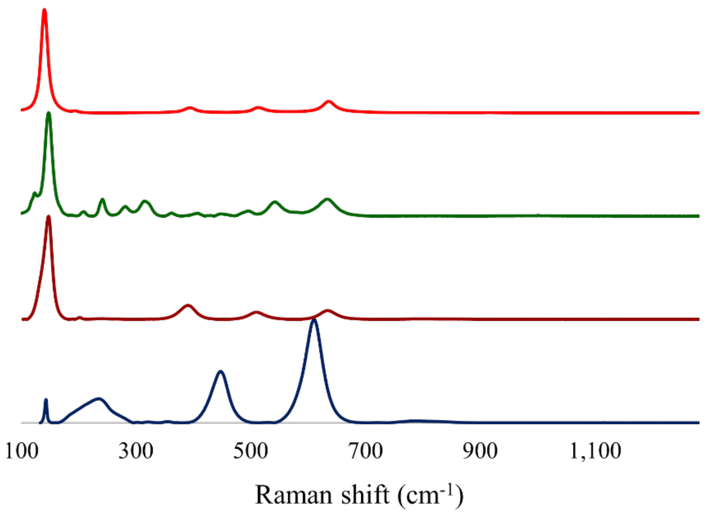

Figure S4.2. Raman spectra overlay of rutile (navy blue), anatase (dark red), and brookite (dark green) standards, and the TiO_2 particles employing the H_2O:NaOH/Heptane system and calcinated at 400°C (red).

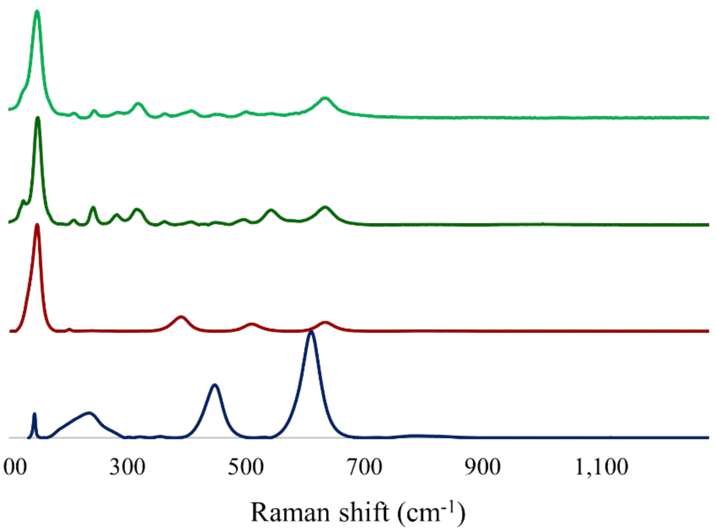

Figure S4.3. Raman spectra overlay of rutile (navy blue), anatase (dark red), and brookite (dark green) standards, and the TiO_2 nanoparticles employing the H_2O:IPA/Heptane system and thermally treated at 200°C (green).

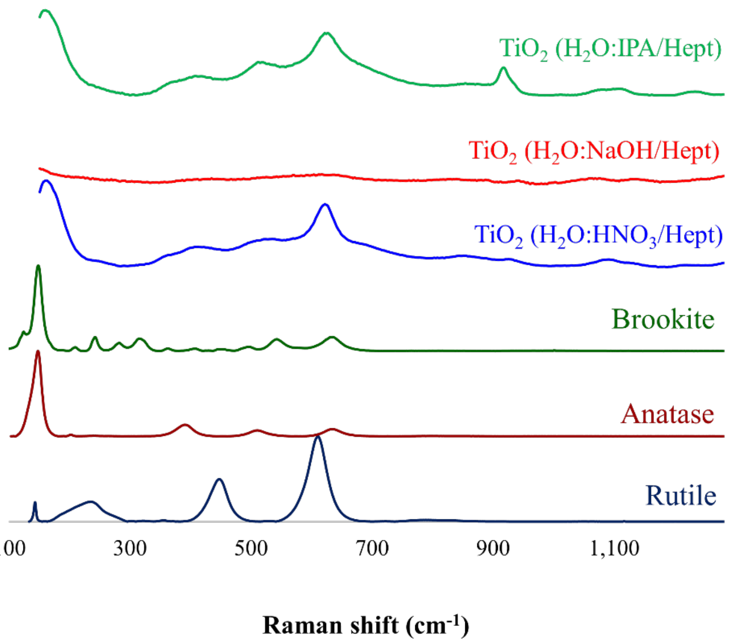

Figure S4.4. Raman spectra overlay of simulated rutile (navy blue), anatase (dark red), and brookite (dark green) phases, with the spectra of the TiO_2 particles synthesized through the PIT-nano-emulsion method without thermal treatment, employing H_2O:HNO_3/Heptane (blue), H_2O:NaOH/Heptane (red), and H_2O:IPA/Heptane (green) emulsion systems.

5. Micro-powder X-ray Diffraction (PXRD)

Figures S5.1-S5.3 depict an overlay of the experimental powder pattern of the TiO_2 particles obtained through the PIT-nano-emulsion synthesis and thermally treated, with the spectra of the three standards rutile (ICSD 165920)[2], anatase (ICSD 154601)[3], and brookite (ICSD 154605)[3]. Figure S5.4. depict an overlay of the experimental powder pattern of the TiO_2 particles obtained through the PIT-nano-emulsion method without being thermally treated. Figure S5.5 depict a comparison between overlayed experimental powder pattern of TiO_2 particles obtained employing H_2O:IPA/Heptane and H_2O:NaOH/Heptane, both calcinated at 200°C. Figure S5.6. depict a comparison between overlayed experimental powder pattern of TiO_2 particles obtained employing H_2O: HNO_3/Heptane and H_2O:NaOH/Heptane, both calcinated at 400°C.

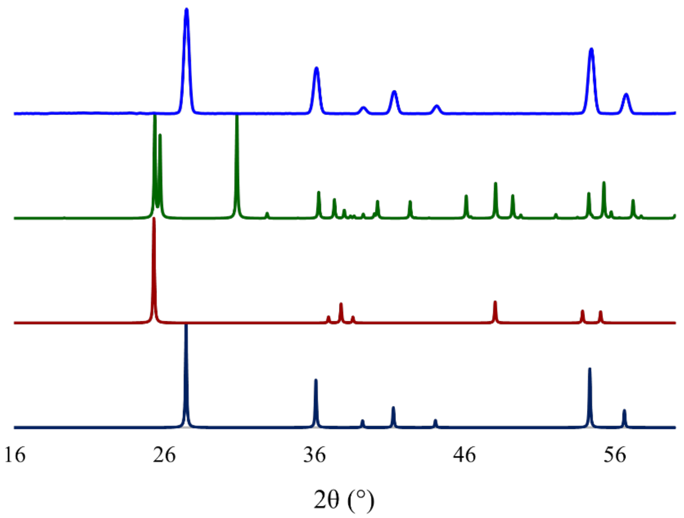

Figure S5.1. Powder X-ray diffractogram overlay of rutile (ICSD 165920, navy blue)[2], anatase (ICSD 154601, dark red)[3], and brookite (ICSD 154605, dark green)[3] standards, and the TiO_2 particles employing the H_2O:HNO_3/Heptane system and calcinated at 850°C (blue).

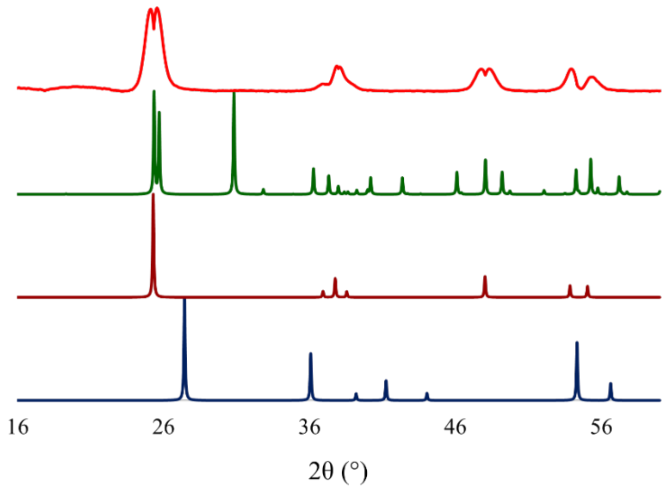

Figure S5.2. Powder X-ray diffractogram overlay of rutile (ICSD 165920, navy blue)[2], anatase (ICSD 154601, dark red)[3], and brookite (ICSD 154605, dark green)[3] standards, and the TiO_2 particles employing the H_2O:NaOH/Heptane system and calcinated at 400°C (red).

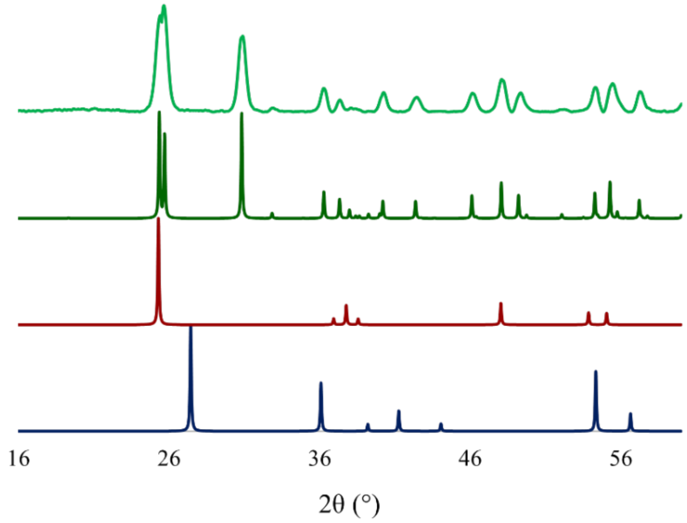

Figure S5.3. Powder X-ray diffractogram overlay of rutile (ICSD 165920, navy blue)[2], anatase (ICSD 154601, dark red)[3], and brookite (ICSD 154605, dark green)[3] standards, and the TiO_2 nanoparticles employing the H_2O:IPA/Heptane system and thermally treated at 200°C (green).

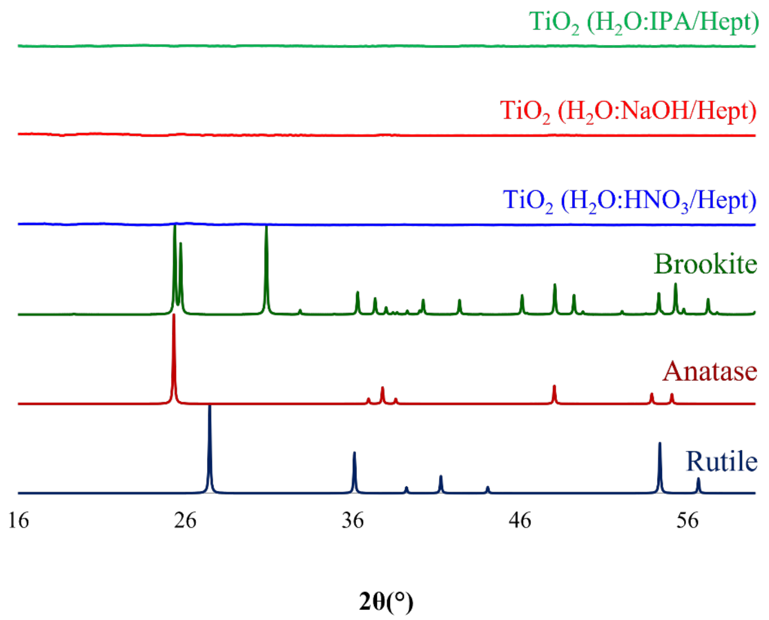

Figure 5.4. PXRD overlay of simulated rutile (ICSD 165920, navy blue)[2], anatase (ICSD 154601, dark red)[3], and brookite (ICSD 154605, dark green)[3] phases, with the diffractograms of the TiO_2 particles synthesized through the PIT-nano-emulsion method without thermal treatment, employing H_2O:HNO_3/Heptane (blue), H_2O:NaOH/Heptane (red), and H_2O:IPA/Heptane (green) emulsion systems.

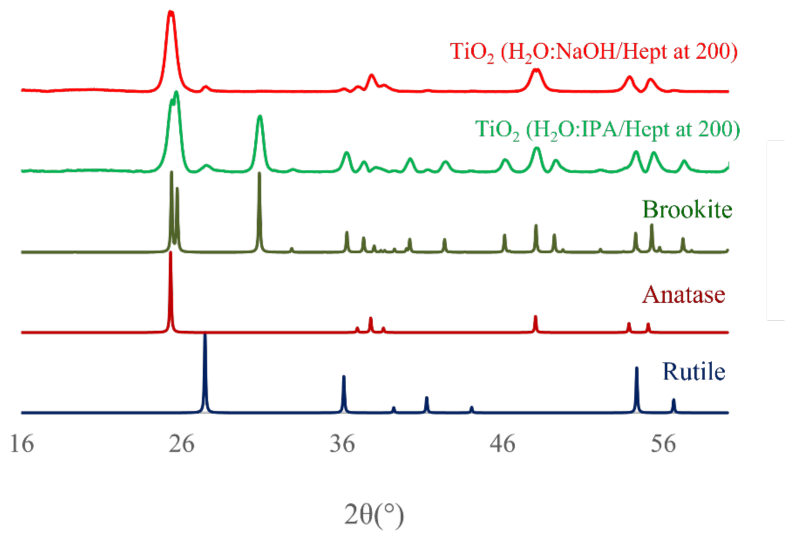

Figure S5.5. Powder X-ray diffractogram overlay of rutile (ICSD 165920, navy blue)[2], anatase (ICSD 154601, dark red)[3], and brookite (ICSD 154605, dark green)[3] standards, and the TiO_2 particles employing the H_2O:IPA/Heptane (green) and H_2O:NaOH/Heptane (red) systems, both calcinated at 200°C.

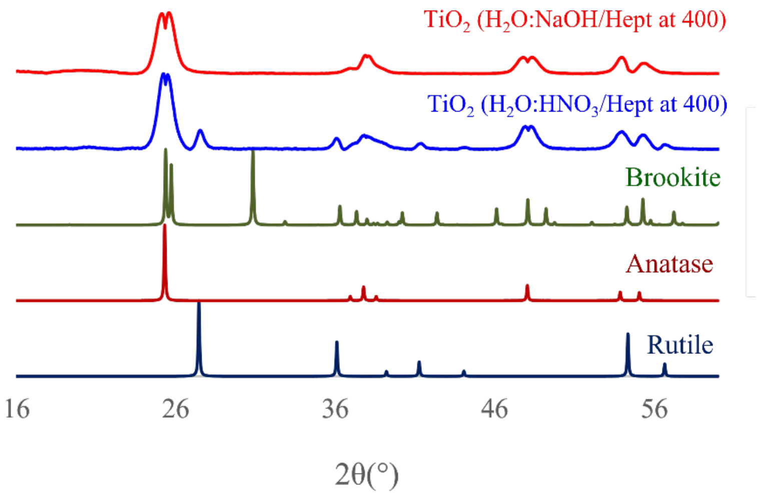

Figure S5.6. Powder X-ray diffractogram overlay of rutile (ICSD 165920, navy blue)[2], anatase (ICSD 154601, dark red)[3], and brookite (ICSD 154605, dark green)[3] standards, and the TiO_2 particles employing the H_2O:HNO_3/Heptane (blue) and H_2O:NaOH/Heptane (red) systems, both calcinated at 400°C.

6. Comparison of Experimental Conditions for TiO_2 Brookite Syntheses

Table S6.1 summarizes the experimental conditions and parameters employed during the brookite-phase oriented PIT-nano-emulsion synthesis as an example, and compared with other reported methods.[1,4,5]

Table S6.1. Comparison of the experimental conditions and parameters employed to isolate brookite phase TiO_2 nanoparticles among the PIT-nano-emulsion synthesis and other reported methods.

Methods	Starting materials	Total quantity of reagents	Synthesis temperature	Synthesis time	Thermal treatment temperature	Thermal treatment time
PIT-nano-emulsion synthesis	Water, Heptane, Brij L4®, titanium (IV) isopropoxide, IPA, glycolic acid, NH_4OH	0.9-5 mL	35-70°C	75 min	200°C	20 h
Mamakhel, *et. al.*[1]	Water, IPA, [titanium (IV) isopropoxide, -butoxide, -tetrachloride, -oxysulfate], glycolic acid, NH_4OH	5-50 mL	90-200°C	25 h	200°C	20 h
Kolesnik, *et. al.*[4]	Water, lactic acid, titanium (IV) butoxide, urea, EtOH	10-60 mL	180°C	48 h	180°C	48 h
Pottier, *et. al.*[5]	Titanium (IV) tetrachloride, HCl, $HClO_4$, NaCl, HNO_3	1-150 mL	100°C	48 h-1 month	100°C	48 h-1 month

7. Size Comparison of the Synthesized TiO_2 Particles by Different Methods

Table S7.1 summarizes the particle size of the experimental rutile, anatase and brookite phase TiO_2 particles obtained from the PIT-nano-emulsion syntheses, determined by scanning electron microscopy (SEM), DLS, as well as the resulting crystallite size determined the Debye-Scherrer equation.

Table S7.1. Comparison of the average particle size of the products before and after calcination of the TiO_2 particles as determined by DLS and SEM-EDS as well as the resulting crystallite size determined using the Debye-Scherrer equation.

Resulting Polymorph	Average particle size by DLS (d.nm)	Average particle size by SEM (nm)	Average crystallite size determined by the Debye-Scherrer Equation		
			2θ (°)	τ (nm)	$\tau_{Average}$ (nm)
Rutile	142.50	~350	27.41	16.75	17.93
			36.22	19.85	
			41.44	16.43	
			54.52	18.67	
Anatase	465.00	~480	25.39	9.8	10.09
			38.06	7.8	
			47.83	8.4	
			54.09	14.34	
Brookite	67.61	~75	25.74	9.57	14.14
			30.97	15.95	
			36.44	17.13	
			40.40	14.50	
			48.28	13.57	

Abbreviations: 2θ (Bragg reflection, °), τ (crystallite size, nm), $\tau_{Average}$ (average crystallite size, nm).

8. References

(1) Mamakhel, A.; Yu, J.; Søndergaard-Pedersen, F.; Hald, P.; Iversen, B. B. Facile Synthesis of Brookite TiO2nanoparticles. *Chem. Commun.* **2020**, *56* (95), 15084–15087. https://doi.org/10.1039/d0cc06795d.

(2) Murugesan, S.; Kuppusami, P.; Mohandas, E. Rietveld X-Ray Diffraction Analysis of Nanostructured Rutile Films of Titania Prepared by Pulsed Laser Deposition. *Mater. Res. Bull.* **2010**, *45* (1), 6–9. https://doi.org/10.1016/j.materresbull.2009.09.012.

(3) Djerdj, I.; Tonejc, A. M. Structural Investigations of Nanocrystalline TiO2 Samples. *J. Alloys Compd.* **2006**, *413* (1–2), 159–174. https://doi.org/10.1016/j.jallcom.2005.02.105.

(4) Kolesnik, I. V.; Kozlov, D. A.; Poluboyarinov, A. S.; Garshev, A. V.; Ivanov, V. K. Non-Classical Growth of Brookite Nanorods. *CrystEngComm* **2019**, *21* (37), 5673–5681. https://doi.org/10.1039/c9ce00682f.

(5) Pottier, A.; Chanéac, C.; Tronc, E.; Mazerolles, L.; Jolivet, J. P. Synthesis of Brookite TiO2 Nanoparticles by Thermolysis of TiCl4 in Strongly Acidic Aqueous Media. *J. Mater. Chem.* **2001**, *11* (4), 1116–1121. https://doi.org/10.1039/b100435m.
